# Investigating and Annotating the Human Peptidome Profile from Urine under Normal Physiological Conditions

**DOI:** 10.3390/proteomes12030018

**Published:** 2024-06-25

**Authors:** Amr Elguoshy, Keiko Yamamoto, Yoshitoshi Hirao, Tomohiro Uchimoto, Kengo Yanagita, Tadashi Yamamoto

**Affiliations:** 1 Biofluid and Biomarker Center, Kidney Research Center, Graduate School of Medical and Dental Sciences, Niigata University, Niigata 951-2181, Japan; amrelguoshy-bbc@ccr.niigata-u.ac.jp (A.E.); yamamotok-bbc@ccr.niigata-u.ac.jp (K.Y.); yoshitoshi.hirao@oist.jp (Y.H.); kyonami-bbc@ccr.niigata-u.ac.jp (T.U.); yanagita-bbc@ccr.niigata-u.ac.jp (K.Y.); 2 Department of Biotechnology, Faculty of Agriculture, Al-Azhar University, Cairo 11651, Egypt; 3 Department of Clinical Laboratory, Shinrakuen Hospital, Niigata 950-2087, Japan

**Keywords:** peptidomics, peptidases, biomarker discovery, urine

## Abstract

Examining the composition of the typical urinary peptidome and identifying the enzymes responsible for its formation holds significant importance, as it mirrors the normal physiological state of the human body. Any deviation from this normal profile could serve as an indicator of pathological processes occurring in vivo. Consequently, this study focuses on characterizing the normal urinary peptidome and investigating the various catalytic enzymes that are involved in generating these native peptides in urine. Our findings reveal that 1503 endogenous peptides, corresponding to 436 precursor proteins, were consistently identified robustly in at least 10 samples out of a total of 19 samples. Notably, the liver and kidneys exhibited the highest number of tissue-enriched or enhanced genes in the analyzed urinary peptidome. Furthermore, among the catalytic types, CTSD (cathepsin D) and MMP2 (matrix metalloproteinase-2) emerged as the most prominent peptidases in the aspartic and metallopeptidases categories, respectively. A comparison of our dataset with two of the most comprehensive urine peptidome datasets to date indicates a consistent relative abundance of core endogenous peptides for different proteins across all three datasets. These findings can serve as a foundational reference for the discovery of biomarkers in various human diseases.

## 1. Introduction

Peptidomics is a part of modern proteomics, by which all endogenous peptides present in biological samples of interest can be identified and characterized [1,2,3]. Although there is not a precise distinction between endogenous peptides and proteins, a commonly accepted characterization of endogenous peptides is that they typically range from dipeptides (consisting of two amino acids) to small proteins with a molecular mass of no more than 20 kDa [2]. They are cleaved from their intact precursor proteins by proteases in the context of post-translation modifications (PTMs) or proteolysis [2]. Endogenous peptides related to PTMs have a diversity of biological and regulatory functions; they act as hormones, cytokines, neuropeptides, and growth factors [4]. Additionally, intracellular and extracellular proteins are subjected to proteolysis, which is required for cell hemostasis, in which the balance between protein synthesis and protein turnover should be kept [2,3]. Two main processes are responsible for intracellular protein turnover, the ubiquitin–proteasome system (UPS), which is considered the largest protein disposal system [5], and autophagy, in which the proteins targeted to lysosomes for degradation by the action of enzymes [6]. Approximately 80% of protein degradation in eukaryotic cells is managed by the UPS, highlighting its predominant role in protein turnover and maintaining cellular protein homeostasis [7]. In both UPS and autophagy, cysteine, aspartate, and threonine proteinases are the main active intracellular endopeptidases at acidic pH levels. In contrast, at a neutral pH, metalloproteinases and serine proteases are the active endopeptidases in the extracellular matrix that facilitate the degradation of extracellular proteins [8,9]. The deregulation of all of these intracellular and extracellular proteases can promote, or sometimes initiate, disease onset and progression. The fragments released from precursor proteins by the action of these regulated proteases mirror the current health status of the organism; this change can be used as a signature for pathological processes governing disease onset and progression in different tissues and biofluids.

Urine, one of the most valuable biological fluids, is composed of a varied set of proteins and peptides. The majority of them are derived from the kidney and urinary tract, which enable deep and comprehensive insight into disease processes affecting the kidney and the urogenital tract, whereas the minority which come from systemic circulation to some extent reflect systematic physiology [10]. Additionally, urine, unlike other body fluids such as blood, can be collected in large volumes non-invasively. Moreover, the urinary peptidome has a relatively high stability because it was thought that proteolytic degradation was completed by the time of voiding. In other words, urine constitutes the end-products of proteolytic degradation, which represent the final snapshot of the peptidome without any subsequent degradation [11].

Despite the growing interest in urinary biomarkers, the exploration of the urinary peptidome remains comparatively underexplored. While numerous studies have focused on the urinary proteome and its implications for disease diagnosis and monitoring [12,13,14,15,16], the specific composition, dynamics, and functional significance of the urinary peptidome have received less attention in both health and disease states [17,18].

One notable advancement in this field is the “CKD273 classifier,” a well-validated panel of 273 urinary endogenous peptides. This classifier aids in diagnosing and predicting the progression of chronic kidney disease (CKD). It is used in routine clinical practice for early detection and risk stratification, demonstrating the potential of the urinary peptidome in improving clinical outcomes for CKD patients [19].

Understanding the urinary peptidome under normal physiological conditions is essential for uncovering novel biomarkers, elucidating disease mechanisms, and advancing personalized medicine approaches tailored to individual patient profiles.

In this context, our study aims to comprehensively characterize the human urinary peptidome under normal physiological conditions. To achieve this, we collected voided urine samples from 19 healthy volunteers with no significant diseases. We then extracted endogenous peptides and performed liquid chromatography–tandem mass spectrometry (LC-MS/MS) analysis, followed by bioinformatic analysis.

A key component of our bioinformatic analysis was the development and use of a Perl script to align urinary endogenous peptides with their corresponding precursor proteins. This script facilitated the assignment of start and end positions of peptides on their precursor proteins and the extraction of octapeptide sequences bordering the N and C termini of the identified endogenous peptides. The output generated by this script served as the foundation for all subsequent analyses, including sequence logo construction using the WebLogo tool, prediction of peptidases involved in peptide generation using Proteasix, and manual validation of peptidase predictions with MEROPS data.

By employing these techniques and leveraging the Perl script for detailed peptide analysis, we aim to identify the composition, origin, and proteolytic processing mechanisms of urinary peptides. Our findings hold promise for uncovering new insights into renal and urogenital physiology, identifying novel biomarkers for disease diagnosis and monitoring, and advancing our understanding of peptide-mediated signaling pathways in health and disease.

## 2. Materials and Methods

### 2.1. Sample Collection and Native Peptide Purification

1. Protein preparation

Voided urine samples were collected from 19 healthy volunteers with no significant diseases (Table S1) by health check at Shinrakuen Hospital according to the urine collection guide proposed by the HUPO Human Kidney and Urine Proteome Project (HKUPP) [20]. One ml of urine each was used for protein preparation by the methanol/chloroform precipitation method as described previously [21]. The supernatant, which contains small molecules such as endogenous peptides, was then processed further.

2. Filtration

The supernatant was filtered through a molecular weight cut-off (MWCO) column with a 30 kDa cutoff (Pall, NY, USA). Before applying the sample to the MWCO membrane filter, the filter was washed with 1 mL of Milli-Q water (MQ) and centrifuged at 3000× *g* for 5 min. Following this, the sample was applied to the MWCO filter and centrifuged at 2000× *g* for 10 min. This final step was repeated twice for thorough filtration.

3. Ethanol Precipitation

The MWCO filtration was performed either before (protocol-1) or after (protocol-2) ethanol precipitation. For the ethanol precipitation step, 20 mL of ethanol was added to the sample and kept at −20 °C for 12 h. The sample was then centrifuged at 3000× *g* for 10 min. The supernatant was discarded, and the pellet was resuspended in 1 mL of 8 M urea/50 mM Tris-HCl (pH 8.0) and mixed well.

4. Reduction and alkylation

Following ethanol precipitation, the reduction was performed by adding 10 µL of 1 M dithiothreitol (DTT) to the sample and incubating at room temperature for 1 h. Alkylation was then conducted by adding 40 µL of 500 mM iodoacetamide at room temperature for 1 h in the dark. The alkylation reaction was quenched by adding 2 µL of 1 M DTT.

5. Peptide Purification

The endogenous peptides generated either from protocol-1 or protocol-2 after reduction and alkylation were subjected to a C18 Monospin column (GL Science, Tokyo, Japan) to purify the peptides. The native peptides were then suspended in 0.1% formic acid. Their concentrations were measured using a NanoDrop 1000 spectrophotometer (Thermo-Fisher, Wilmington, DE, USA) and stored at −80 °C until use.

### 2.2. LC-MS/MS, Mass Spectrometric Analysis

One sample of 1μg native urine peptides each was analyzed twice on an ultrahigh-pressure nanoflow chromatography system (nanoElute, Bruker Daltonics, Billerica, MA, USA) coupled to a trapped ion mobility quadrupole time-of-flight mass spectrometer (timsTOF Pro, Bruker Daltonics) in PASEF mode. Peptides were separated on an analytical column (25 cm × 75 μm, C18, 1.6 μm, Aurora Column, Ion Opticks, Victoria, Australia), at a flow rate of 400 nL/min using a 120 min gradient with a liner increase of acetonitrile to 37% (mobile phase A: water with 0.1% formic acid; mobile phase B: acetonitrile with 0.1% formic acid) at a 50 °C column oven temperature.

The eluting peptides were interrogated by an MS acquisition method recording spectra from 100 to 1700 *m*/*z* and ion mobility scanned from 0.6 to 1.6 Vs/cm^2^ over a ramp time of 100 ms. Data-dependent acquisition was performed using 10 cycles of PASEF MS/MS per total cycle time of 1.1 s with a target intensity of 20 k. A polygon filter was applied within the ion mobility over *m*/*z* heatmaps to exclude low-*m*/*z*, singly charged ions from PASEF precursor selection. An active exclusion time of 0.4 min was applied to precursors that reached 20,000 intensity units. Precursors for data-dependent acquisition were fragmented with an ion mobility-dependent collision energy, which was linearly increased from 20 to 59 eV.

### 2.3. Peptide Quantification and Identification

The MASCOT search engine [22] (version 2.3.01, Matrix Science) was used to search the acquired spectra against the Swiss-Prot database (release number: 2018-07) (downloaded on 31 July 2018) alongside the contaminant database. Also, searching against the decoy database was considered to calculate FDR. The search parameters were set to no enzyme specificity, carbamidomethyl cysteine was used for fixed modification, and no variable modifications were assigned. The mass tolerance was set to 50 ppm and 0.05Da for MS and MS/MS, respectively.

Our strategy involves a two-step filtration process for endogenous peptide identifications to maximize the retention of true positive peptides. Initially, we apply a relatively loose false discovery rate (FDR) threshold of 5% at the peptide–spectrum match (PSM) level, rather than a stricter threshold of 1%. This allows us to capture a broader set of peptide identifications, including those that might be excluded under a more stringent criterion.

In the second filtration step, we refine our selection by retaining only those peptides identified in at least 50% of the samples. This ensures that we focus on peptides that are consistently detected across the dataset, thus increasing the likelihood that they are true positives. By initially using a 5% FDR threshold, we retain peptides that may have slightly higher FDRs but demonstrate reproducibility across multiple samples. This approach helps us avoid the premature exclusion of potentially significant peptides that might be lost if we start with a strict 1% FDR threshold.

Relative abundance was used as a metric for peptide quantification where the spectral count of each peptide was divided by the sum of all endogenous peptides in a certain sample.

### 2.4. The Origin of the Degraded Proteins Associated with the Identified Endogenous Peptides

The human RNA expression pattern dataset “proteinatlas.tsv.zip” was downloaded from the Human Protein Atlas [23] (HPA, version 23) website. It provides comprehensive insights into the RNA expression profiles of human genes across 37 distinct human tissues. It was used to examine the origin of the precursor proteins, generating these endogenous peptides.

Within the HPA framework, RNA expression patterns of putative protein-coding genes were classified with regard to their specificity across all major organs and tissue types into five categories: (1) those have elevated mRNA expression level in a particular tissue, at least 5-fold compared with other tissues (tissue enriched genes); (2) those have elevated mRNA expression level in a group of 2–7 tissues, with at least 5-fold (group enriched genes), (3) those have elevated expression level in a particular tissue, with at least 5-fold compared with average levels in all tissues (tissue enhanced genes), (4) those with low tissue specificity, and finally (5) those that are not detected in any tissue (not detected).

Furthermore, regarding distribution, the RNA expression patterns of putative protein-coding genes are categorized into five distinct classes: (1) those present exclusively in one tissue (Detected in single), (2) those detected in a limited number of tissues, more than one but less than one-third (Detected in some), (3) those present in at least one-third but not all tissues (Detected in many), (4) those ubiquitously expressed across all tissues (Detected in all).

Simultaneously, an investigation into the tissue type distribution was conducted for enriched or enhanced protein-coding genes, shedding light on the tissues contributing most significantly to urinary endogenous peptide generation.

### 2.5. Cleavage Site Analysis of Urinary Peptides

#### 2.5.1. Determination of Cleavage Site Sequences Adjacent to Precursor Endogenous Peptides

A Perl script (Texts S1 and S2) was developed to align urinary endogenous peptides with their corresponding precursor proteins, facilitating the assigning of the start and end position of the peptide on its precursor protein, and the extraction of octapeptide sequences bordering the N and C termini of the identified endogenous peptides. The output data generated from this script is extremely important because it will act as input for further processing done in the next steps.

#### 2.5.2. Construct Sequence Logos

WebLogo tool [24] was employed to construct sequence logos representing the relative occurrence of each amino acid at specific positions within the extracted N and C-terminal cleavage site octapeptides from the previous step, offering a visual depiction of sequence conservation and variability.

#### 2.5.3. Prediction of Peptidases Involved in Peptide Generation

We used the peptide start and end positions on their precursor proteins obtained from the Perl script in step 5.1 with peptide sequence and protein header as input for Proteasix [25] a peptide-centric computational tool, to predict the peptidases responsible for generating endogenous peptides. Proteasix operates as a web-based platform specifically designed to analyze proteolytic events contributing to the natural generation of peptides. It identifies both observed and predicted proteases involved in the proteolytic processing of these cleavage sites across three prominent species: *Homo sapiens, Mus musculus,* and *Rattus norvegicus.*

#### 2.5.4. Validation of Proteasix Predictions through Manual Verification and Leveraging MEROPS Data

To confirm the predictions made by Proteasix, manual confirmation was performed, drawing upon data from the MEROPS database (Release number: 12.3) [26]. MEROPS serves as a meticulously curated repository of information about various peptidases and their associated protein inhibitors, providing comprehensive insights into the regulatory mechanisms governing peptidase activity in vivo. This manual validation process enhances the reliability and comprehensiveness of the peptidase prediction outcomes obtained from Proteasix analysis.

### 2.6. Data Analysis and Visualization Tools

The data cleaning, manipulation, and summarization processes were facilitated by the utilization of the Dplyr, tidyr, and stringr libraries. Concurrently, the ggplot library was used to generate all the plots in the manuscript.

## 3. Results

### 3.1. Urine Peptidome Profile

Our dual-step filtration process, beginning with a loose 5% FDR followed by consistency-based selection, allows us to balance the sensitivity and specificity of peptide identification, ensuring a robust set of peptides for subsequent analysis.

**Figure 1:**
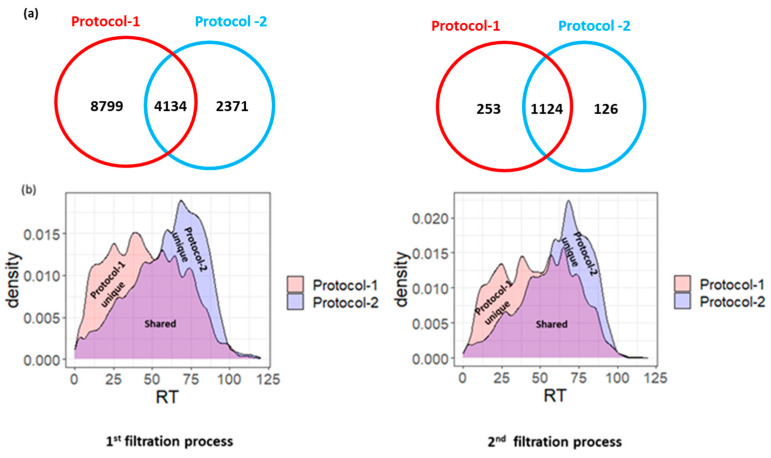
(**a**) Venn diagram showing the overlapped and the exclusive endogenous peptides identified by Protocol-1 and Protocol-2 through the first and second filtration processes, respectively. (**b**) Density plot showing the retention time distribution for the identified endogenous peptides by Protocol-1 and Protocol-2 through the first and second filtration processes, respectively.

In the first step of our filtration process, we successfully identified 29,372 endogenous peptides derived from 7220 precursor protein groups in 19 samples (Table S2). These identifications were obtained from the Mascot search engine at 5% PSM FDR. Specifically, 18,485 peptides were uniquely identified using the molecular weight cutoff (MWCO) filtration step before ethanol precipitation (protocol-1), 6367 were identified using the molecular weight cutoff (MWCO) filtration step after ethanol precipitation (protocol-2) (as described in the materials and methods section), and 4520 peptides were common to both methods (Figure 1a) (Table S2).

It is noteworthy that the observed increase in peptide identification with protocol-1 compared to protocol-2 likely stems from the more efficient enrichment and concentration of peptides achieved by conducting filtration after methanol/chloroform precipitation. This sequence maximizes peptide retention while minimizing losses, resulting in a higher yield of peptides available for subsequent analysis and identification. Conversely, applying ethanol precipitation before MWCO filtration may lead to the inefficient co-precipitation of smaller hydrophilic peptides, resulting in their potential loss.

Overall, these protocols collectively facilitate the generation of a comprehensive peptidome profile, capturing a diverse array of peptides with varied hydrophilic and hydrophobic characteristics (Figure 1b).

In the second filtration process, we focused only on the endogenous peptides identified in at least ~50% of samples (10 samples), as they more comprehensively reflect the profile of a healthy urine peptidome. This subset comprises 1505 endogenous peptides corresponding to 436 precursor proteins (Table S3).

Of note, when we employed a stricter 1% FDR threshold in the first step, we identified 15,304 endogenous peptides, which further decreased to 953 peptides after the second filtration. This comparison indicates that starting with a strict cutoff value of 1% FDR led to the loss of 550 true positive peptide identifications. Notably, these lost peptides tend to be identified at an FDR of less than 2%.

Additionally, peptides “AEDEGGEE” and “GVPHGKGRAIRLGVLKSPLKKLMSTA” were removed from the final list in Table S3. These peptides were identified across a wide range of retention times and, upon re-evaluation, the majority of spectra matching these peptides showed slightly higher FDR values. Consequently, the final list of endogenous peptides used for further analysis consisted of 1503 peptides (Table S3).

### 3.2. Consistency-Based Selection

**Figure 2:**
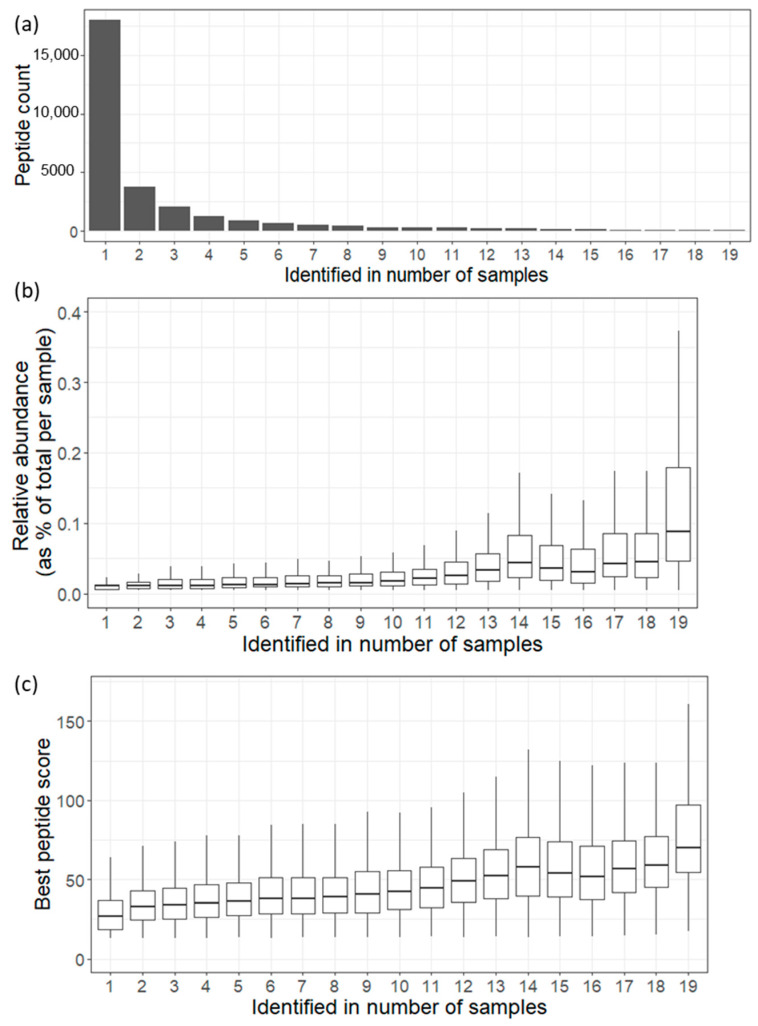
(**a**) Bar plot showing the urinary endogenous peptides identified in number of samples. (**b**). Boxplot showing the relative abundance distribution for the urinary endogenous peptides identified in number of samples. (**c**). Boxplot showing the best ion score distribution for the urinary endogenous peptides identified in number of samples.

Regarding the identified endogenous peptides in a number of samples, our data showed that out of the total 29,372 identified endogenous peptides, a mere 69 peptides, constituting only 0.23% of the entire urinary endogenous peptide count, were consistently detected in all 19 analyzed urine samples, forming what is referred to as the core peptidome (see Figure 2a). These peptides, originating from 26 proteins, represent a series of fragment cascades with slight amino acid variations and are characterized as medium-to-high-abundance peptides. Despite their small proportion of the total peptidome count, they contribute to approximately 10% of the overall quantified peptidome, as indicated in Table S4.

This finding underscores the dynamic and diverse nature of the urinary peptidome, where only a fraction of peptides appear to be consistently present across a heterogeneous population. The presence of a core peptidome suggests the existence of fundamental biological processes or molecules that are shared among individuals, reflecting essential aspects of urinary physiology or metabolism.

However, the vast majority of identified peptides exhibit variability in their presence or abundance across samples, highlighting the complexity and heterogeneity of the urinary peptidome within and between individuals. Factors such as genetic variation, physiological status, diet, and environmental influences may contribute to this observed diversity.

### 3.3. Peptide Characteristics and Distribution

Notably, as depicted in Figure 2b,c, the peptide spectral count and the best ion score distributions follow a certain pattern where the endogenous peptides detected in more samples tend to be assigned to more spectra with a better score. For instance, the core endogenous peptides, identified in all samples, show a tendency to be assigned the highest number of spectra (median = 10, interpercentile range = 14 (5:19)) with a distribution of the best peptide scores (median = 70.18, interpercentile range = 43.41 (54.62:98.03)).

The top three precursor proteins contributing to the peptidome signal are uromodulin, prostaglandin-H2 D-isomerase, and serum albumin, representing approximately 7.2%, 4.5%, and 3.0% of the peptidome signal, respectively.

### 3.4. The Origin of the Urinary Endogenous Peptides

**Figure 3:**
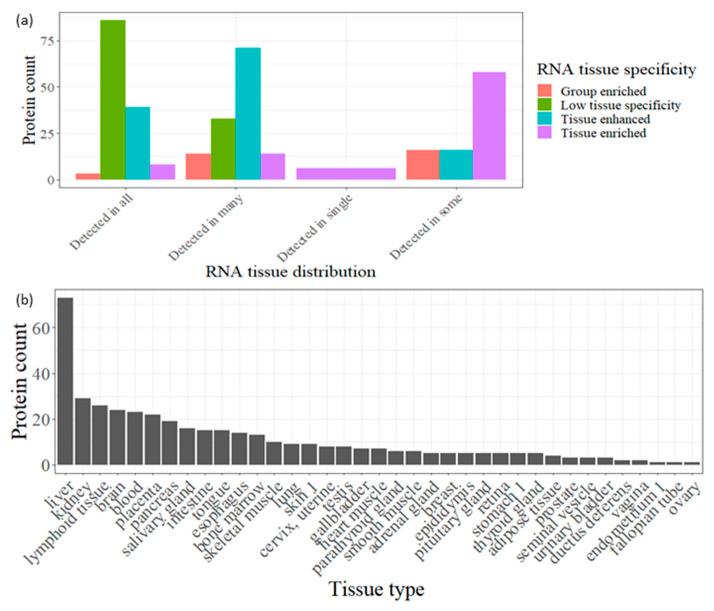
(**a**) Bar plot showing the RNA tissue distribution for the degraded proteins (correspond to the detected peptides in at least in 10 samples) broken up by RNA tissue specificity. (**b**). Bar plot showing the count of degraded proteins (correspond to the detected peptides in at least 10 samples) which enriched or enhanced in certain tissue according HPA data in descending order.

The Human Protein Atlas data were utilized to investigate the origin of the 436 degraded proteins associated with the 1503 identified endogenous peptides across various human tissues. Our analysis revealed that approximately 27% of these proteins exhibit low tissue specificity, indicating a high likelihood of originating from multiple tissues and ultimately being present in urine (Figure 3a). In contrast, the remaining 73% of the degraded proteins found in urine tend to display high specificity for particular tissues or groups of tissues, categorizing them as enriched and enhanced proteins. Notably, the liver stands out with the highest number of tissue-enriched or enhanced genes (73), followed by the kidney (29) (Figure 3b). This finding underscores the significance of utilizing urine as a biofluid for biomarker discovery in liver and kidney diseases.

### 3.5. Cleavage Site Analysis of Urinary Peptides

**Figure 4:**
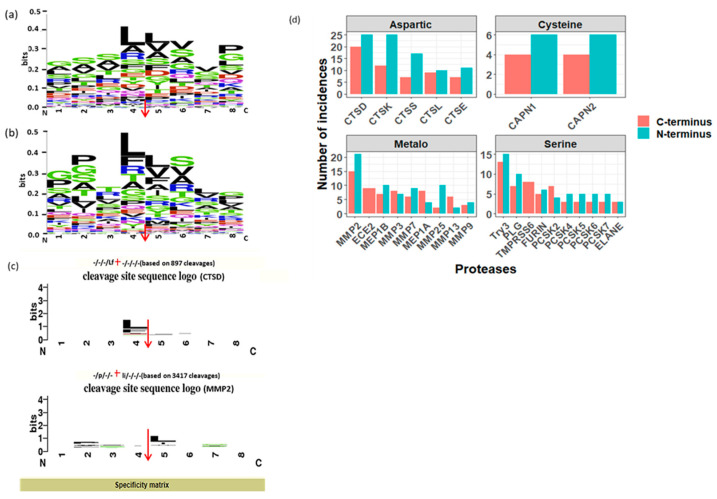
(**a**,**b**). Sequence logo of N- and C termini of urinary endogenous peptides, respectively detected in at least 10 samples using weblogo. (**c**). Specificity matrix showing the cleavage site sequence logo of CTSD and MMP2 proteases, respectively. The red arrows are representing the cleavage site in figures (**a**–**c**). (**d**). Bar plot showing the distribution of proteases involved in the generation of urinary endogenous peptides (detected at least in 10 samples) facetted by the proteases catalytic type.

To investigate the proteolytic mechanisms associated with normal physiological conditions, firstly, we examined the cleavage sites associated with the N and C termini of urinary peptides generated from our bioinformatics tool, and subsequently visualized by WebLogo tool. Notably, the octapeptide profile (Figure 4a,b) revealed a notable abundance of proline and glycine residues in the vicinity of both the N- and C termini of urinary peptides. This result aligns with a study by Julie et al. study [18], in which a prevalence of proline residues in healthy urinary peptides, particularly at or near the N- and C termini, was observed compared to peptides in diabetic conditions.

Additionally, the predominant motifs for the N and C terminus cleavage sites are “GSSL*LVVP” and “GPTL*LSLG”, respectively, indicating that the peptidases with the highest involvement in urinary endogenous peptide generation exhibit a preference for cleaving before and after leucine (Figure 4a,b).

Subsequently, we employed the Proteasix peptide-centric prediction tool, utilizing the observed mode, to match the N and C-terminus octapeptides cleavage sites flanking the identified endogenous peptides to the proteases cleavage site associations compiled from the literature. This analysis yielded 380 combinations of predicted proteases and cleavage sites.

As depicted in Figure 4d, peptidases of the aspartic and metallopeptidases catalytic types predominated among other catalytic types, collectively responsible for generating 35% and 32% of urinary endogenous peptides, respectively. Notably, CTSD and MMP2 emerged as the most prominent peptidases in the aspartic and metallopeptidases catalytic types, respectively. Conversely, serine and cysteine peptidases contributed approximately 27% and 5%, respectively, to the generation of endogenous urinary peptides, with trypsin-3 (try3) being the over-represented peptidase in the serine catalytic type (Figure 4d).

Manual confirmation of Proteasix predictions, aligning with MEROPS data, demonstrated a high consistency between the patterns in the octapeptide profile and the frequency of predicted proteases. For instance, according to the MEROPS database, CTSD exhibited a pronounced preference for cleaving after leucine (L) (Figure 4c), while MMP2 showed a distinct preference for cleaving before leucine (L) (Figure 4c). Concurrently, the predominant octapeptides for the N and C terminus cleavage sites were “GSSLLVVP” and “GPTLLSLG,”, respectively, underscoring the significant involvement of CTSD and MMP2 in the generation of urinary endogenous peptides.

### 3.6. The Core Degradome Repertoire of Urinary Proteins

The rationale behind investigating these core components lies in their potential role as key mediators or markers of biological processes within the urinary system. These peptides may originate from various sources, including intracellular proteins, extracellular matrix components, or circulating proteins, undergoing proteolytic cleavage and subsequent excretion into the urine. Their consistent presence across multiple samples suggests a degree of biological significance and may hint at underlying regulatory mechanisms governing their generation and clearance.

**Figure 5:**
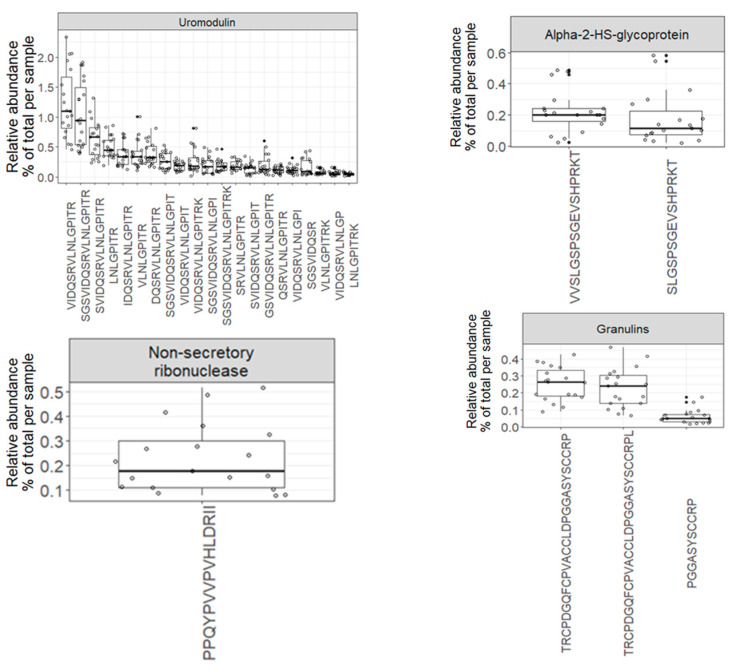

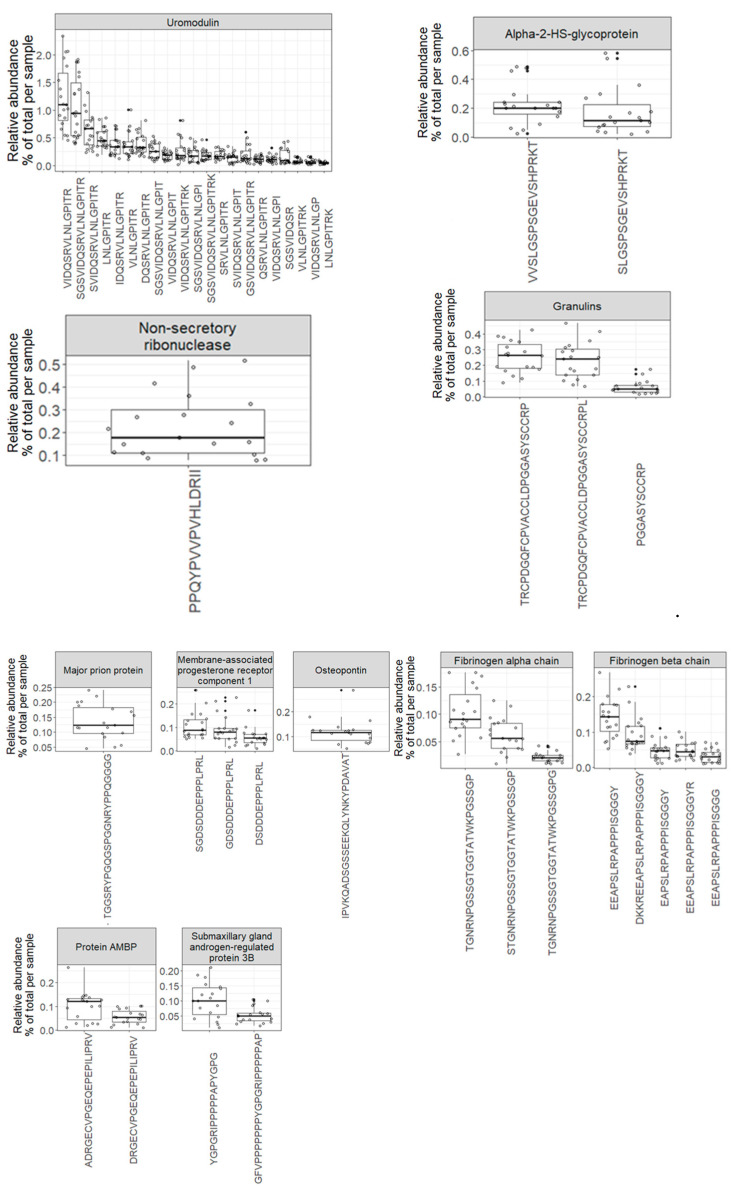

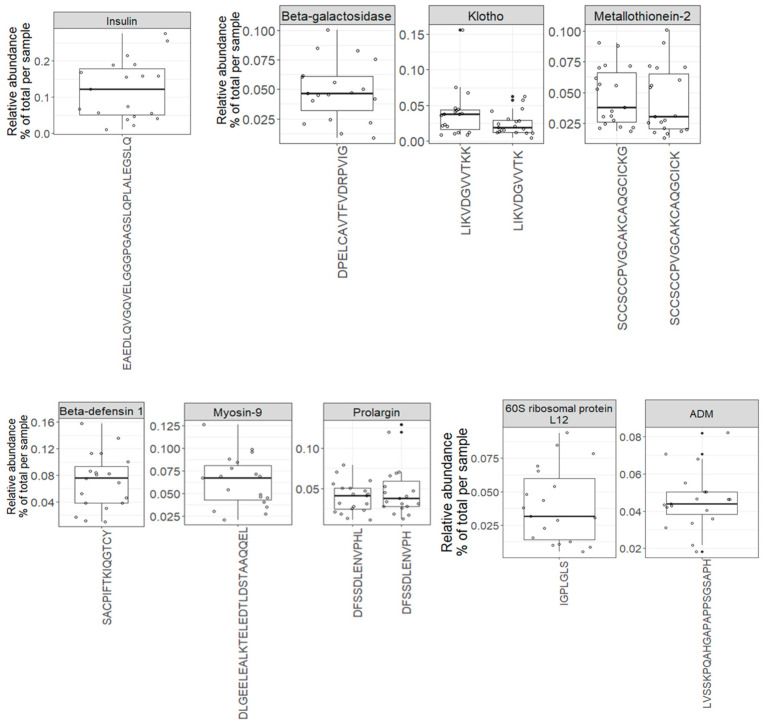
Boxplots and jitter plots showing the distribution of the relative abundance (% of total signal per sample) of 69 core peptides derived from 26 precursor proteins.

The variety of endogenous peptides found in normal urine, known as the degradome repertoire, reflects the typical activity of proteases in normal physiological conditions. Therefore, any alterations in the activity of these proteases during the development and progression of diseases will lead to changes in the degradome repertoire of these urinary proteins. In Figure 5, the relative abundance of 69 core peptides, serving as degradation products for 26 precursor proteins under normal physiological conditions, is depicted (Table S4). For instance, the 21 core endogenous peptides of uromodulin exhibit an interesting distribution, with “VIDQSRVLNLGPITR” and “SGSVIDQSRVLNLGPITR” being the most abundant peptides, constituting approximately 1% and 0.9% of the total peptidome signal, respectively. In contrast, “LNLGPITR” is the least abundant peptide, with a median of around 0.4%. Additionally, the 5 core endogenous peptides of Collagen alpha-1 (XVIII) chain display a distinct pattern, with “DDILASPPRLPEPQPYPGAPHHSS” being the most abundant peptide (median = 0.17) and “DDILASPPRLPEPQPYPGAPH” being the least abundant (median = 0.04).

Since these profiles encompass repeatedly identified and quantified peptides across all samples, they serve as a highly reliable baseline reference for biomarker discovery in various human diseases.

### 3.7. Placing of Findings in the Existent Literature

To assess whether our findings accurately represent the overall urine peptidome profile under normal physiological conditions, we conducted an analysis using two of the most comprehensive urine peptidome datasets available. These datasets, comprising 5011 and 4696 endogenous peptide lists from healthy urine, were obtained from prior studies conducted by Julie et al. [18] and Ashley et al. [17], respectively.

**Figure 6:**
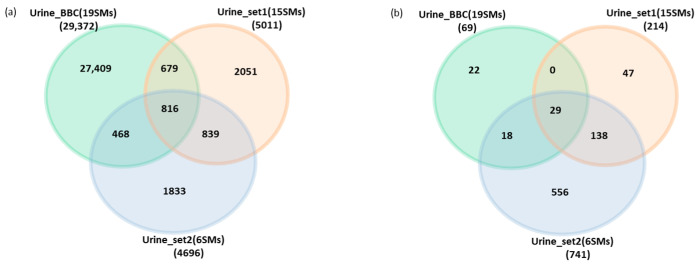
(**a**). Venn diagram showing the overlapped and exclusive endogenous peptides identified by three different human peptidome datasets (Urine_BBC is the dataset represented in this manuscript). (**b**). Venn diagram showing the overlapped and the exclusive core endogenous peptides (detected systematically in all samples) identified by three different human peptidome datasets.

An examination of the overlap and exclusivity of peptide identifications among our dataset and the other two datasets revealed that 816 endogenous peptides are common to all three datasets (Figure 6a). Furthermore, we investigated the overlap and exclusivity of peptides within the core peptidome (peptides systematically identified in all samples) for our dataset and the other two datasets. Our findings indicated that 29 endogenous peptides, originating from 9 precursor proteins, consistently appeared across all samples in all datasets (Figure 6b).

The assessment of the degradome repertoire of these 9 precursor proteins using their released endogenous peptides is crucial, as it signifies the comprehensive degradome profile of these proteins across three different datasets under normal physiological conditions. Subsequently, we examined the signal ratios of these 29 endogenous peptides relative to the total signal of core peptides in specific proteins and samples across the three datasets. Remarkably, the peptide ratios within certain proteins appeared nearly identical across all datasets.

**Figure 7:**
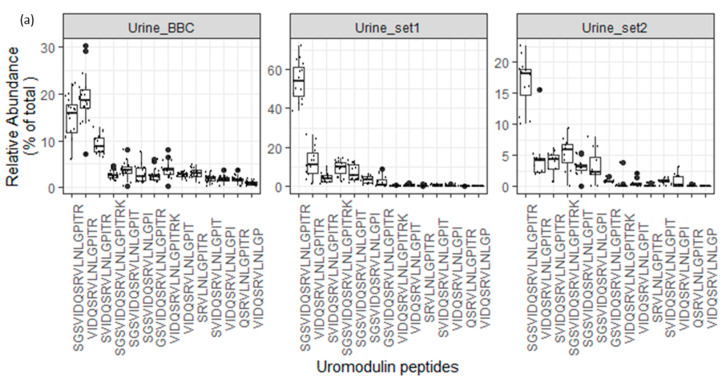

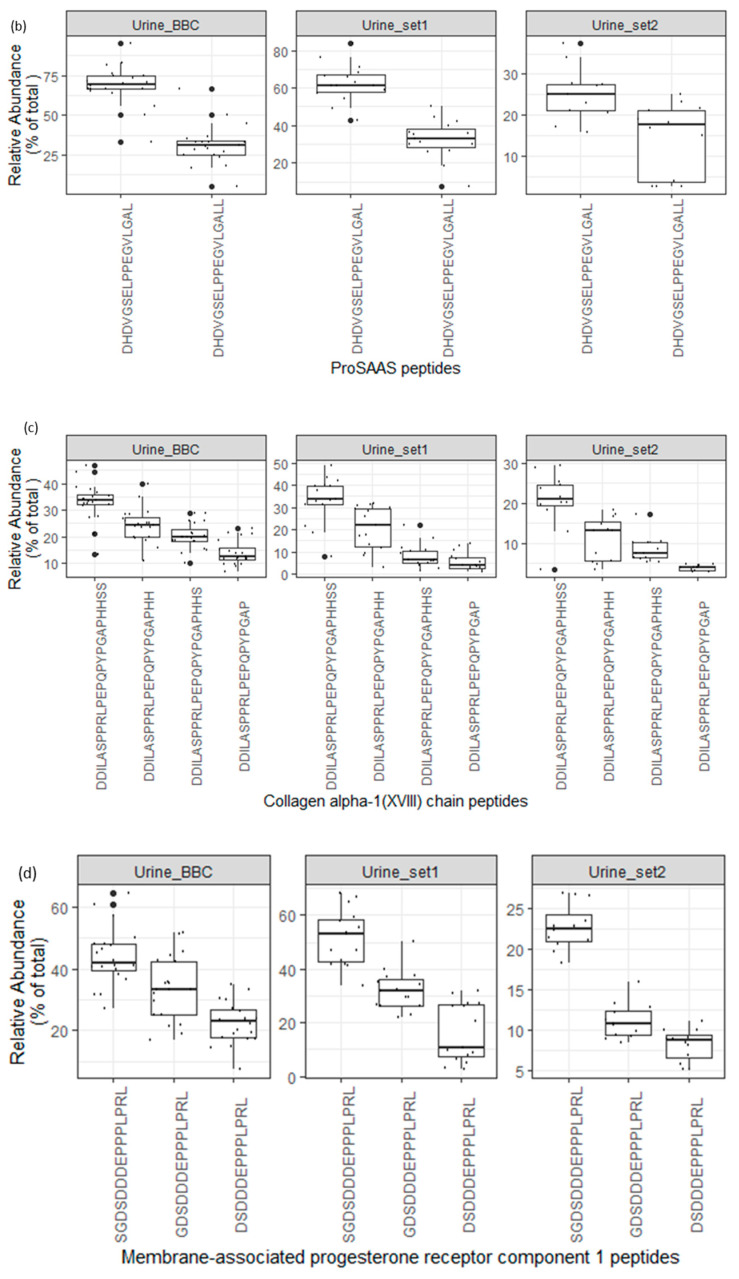
(**a**–**d**). Boxplots and jitter plots showing the relative abundance distribution (% of total core peptide signal per protein in each sample) of some core peptides among 3 different peptidome datasets (Urine_BBC is the dataset represented in this manuscript).

As depicted in Figure 7a, the endogenous peptide “SGSVIDQSRVLNLGPITR” in uromodulin exhibited significantly higher abundance compared to other uromodulin peptides in all analyzed urine datasets. Additionally, as illustrated in Figure 7b–d, core endogenous peptides derived from ProSAAS, collagen alpha-1 (XVIII), and Membrane-associated progesterone receptor component 1 precursor proteins displayed a consistent pattern across all analyzed datasets.

## 4. Discussion

The exploration of the human urinary peptidome under normal physiological conditions is relatively limited compared to studies on the urinary proteome. Our objective was to comprehensively examine the profile of the human urinary peptidome under normal physiological conditions, including their origin, cleavage site patterns, and the involvement of peptidases in these cleavages.

In this investigation, we identified 29,372 endogenous peptides from 19 healthy urine samples. This vast dataset sheds light on the proteoforms present in the urinary peptidome, reflecting the complexity of protein species in urine.

Unlike tryptic peptides in proteomics, endogenous peptides in peptidomics are characterized by low abundance and low detectability rates across samples. For instance, 61% of the urinary peptidome exhibited significant variability among individuals, while approximately 5% were consistently identified in at least 50% of samples, and fewer than 1% were systematically identified in all 19 samples. These findings align with a previous study, indicating that the core peptidome represents about 3% of all peptide identifications in all 15 samples. These findings highlight the proteome complexity and dynamic nature of the urinary peptidome.

The urine peptides and proteins are known for their considerable variability, influenced by a variety of factors such as gender, age, diet, hydration status, physical activity, and circadian rhythms [27,28]. This inherent variability is often higher compared to other biofluids like blood or cerebrospinal fluid, which are more tightly regulated in terms of their protein and peptide composition. Despite this variability, urine offers a non-invasive means of sampling and provides a rich source of peptides that reflect the physiological and pathological state of the body, particularly the renal and urinary systems [27].

Our analysis of 69 core peptides demonstrated consistent relative abundance across multiple urine samples, suggesting a degree of stability that is crucial for their potential use as biomarkers. This consistency, despite the expected variability in the urine peptidome, underscores the robustness of our findings and supports the utility of urine as a viable biofluid for peptidomic studies. Further comparative studies involving other biofluids would be beneficial to fully elucidate the variability and stability of peptidomes across different biological matrices.

Remarkably, around 73% of urinary peptides identified in this study exhibited elevated expression in specific tissues or groups of tissues, with the brain being the most enriched tissue in urine among the 29,372 endogenous peptides identified. This aligns with results from a comprehensive analysis of the normal urinary proteome [29]. However, when considering only peptides identified in at least 50% of samples (more consistent identifications), the liver emerged as the most enriched tissue in urine. Notably, the liver plays a key role in synthesizing plasma proteins and maintaining plasma constituents.

It is noteworthy that only five degraded precursor proteins are tissue-specific, with four being liver-specific (prothrombin, coagulation factor IX, hemopexin, and thyroxine-binding globulin) and one kidney-specific (uromodulin). Changes in the healthy degradome signature of these proteins may provide valuable insights into the onset and progression of diseases related to these tissues.

Analyzing the pattern of octapeptide flanking identified endogenous peptides revealed that proteases with the highest involvement in endogenous peptide generation prefer to cleave between leucines at the N and C-termini. This pattern, occurring systematically in at least 10 out of 19 samples, is considered a representative signature of a healthy endogenous peptidome profile, shedding light on the proteolytic processing events shaping the urinary peptidome.

Predicting the proteases involved in the generation of normal urinary endogenous peptides highlighted the predominance of aspartic, metallopeptidase, serine, and cysteine peptidases in the urine peptidome. This predominance can be attributed to their crucial biological roles and substrate specificities, which align well with the protein and peptide composition of urine.

Aspartic peptidases, such as cathepsin D (CTSD), are vital for lysosomal protein degradation in almost all tissues with higher expression levels in the brain, leading to the breakdown of intracellular proteins and the generation of peptide fragments [30,31]. These by-products are eventually excreted in urine, explaining the significant presence of these peptidases. Similarly, metallopeptidases like matrix metalloproteinase 2 (MMP2) are involved in extracellular matrix (ECM) remodeling and degradation, processes that generate a diverse array of peptide fragments [32].

Notably, the reduced activity of MMP-2 has been associated with kidney damage in diabetic kidney tissue [33].

Serine peptidases, including trypsin-3 (TRY3), play essential roles in digestion, immune response, and blood coagulation [34], leading to the generation of numerous peptide fragments that are excreted in urine. Their broad substrate specificity and involvement in multiple biological pathways account for their significant contribution. Cysteine peptidases, such as calpain 1 (CAPN1) and calpain 2 (CAPN2), are involved in cytoskeletal remodeling and signal transduction, generating stable peptide fragments from structural and signaling proteins [35]. The combined activities of these peptidases reflect the body’s ongoing proteolytic processes, providing a comprehensive snapshot of physiological states and proteolytic activities in the urine peptidome.

Comparing our peptidome profile with the most comprehensive dataset to date returned 816 common endogenous peptides. This variability emerged due to methodological differences between our datasets and other peptidome datasets we compared with as well as other physiological and environmental differences that we previously referred to in the discussion part regarding the variability of urinary peptidome among samples of our study.

Among these, 29 endogenous peptides were consistently detected in all samples across all datasets, indicating high stability and potential functionality. These stable proteoforms could serve as valuable biomarkers for disease states or physiological conditions, warranting further investigation into their role and significance. In a previous study, one of these 29 endogenous peptides, “SGSVIDQSRVLNLG-PITR” (uromodulin peptide) showed decreased expression in diabetes, suggesting its potential relevance in disease states [18].

## 5. Conclusions

The continuous turnover and processing of proteins by various peptidases generate a diverse array of endogenous peptides, which are subsequently excreted in the urine. This process provides a comprehensive snapshot of the body’s proteolytic activities and physiological state. Analyzing the human urinary peptidome under normal physiological conditions offers valuable insights into its complexities, including the identification of peptidome patterns for each precursor protein in the human proteome and the specific proteases involved in their cleavage. Our study contributes significantly to the field through several key findings.

Firstly, our dual-step filtration process, beginning with a loose 5% FDR followed by consistency-based selection, ensures a robust set of endogenous peptides for subsequent analysis. This methodological rigor balances sensitivity and specificity, enhancing the reliability of our findings and providing a valuable reference point for future research. Secondly, by comparing our findings with two of the most comprehensive urine peptidome datasets available, we demonstrated the robustness of our identified peptides. This comparative approach provides additional validation and increases the applicability of our dataset for broader biomarker discovery efforts.

Additionally, we identified 69 core peptides with consistent relative abundance across multiple urine samples. Of these, 29 endogenous peptides from 9 precursor proteins were consistently detected across multiple datasets. This core peptidome indicates a degree of stability crucial for their potential use as biomarkers. The consistency observed, despite the expected variability in the urine peptidome, underscores the robustness of our findings and supports the utility of urine as a viable biofluid for peptidomic studies. Finally, the identified core peptides are likely key components of the urine peptidome, reflecting the underlying physiological state. Researchers can use our dataset as a baseline to compare against pathological conditions, aiding in the identification of potential biomarkers associated with various diseases.

In summary, the urine peptidome profile generated in our current work serves as a reliable and physiologically relevant resource for future biomarker discovery. Researchers can refer to our dataset to identify consistent and robust peptides, compare their findings, and enhance their understanding of the urine peptidome under both normal and pathological conditions.

## Data Availability

The original contributions presented in the study are included in the article/supplementary material, further inquiries can be directed to the corresponding author.

## Supplementary Materials

The following supporting information can be downloaded at: https://www.mdpi.com/article/10.3390/proteomes12030018/s1, Table S1: Characteristics of the 19 samples analyzed in this study, Table S2: Characteristics of 29,372 human urinary endogenous peptides identified across 19 healthy volunteer samples in both protocol-1 and protocol-2, Table S3: Characteristics of 1503 human urinary endogenous peptides identified in at least 10 out all 19 healthy volunteer samples in both protocol-1 and protocol-2, and they are used for investigating the urinary endogenous peptide origin, and proteolytic processing mechanisms underlying urinary peptides, Table S4: The features of the human urinary endogenous peptides detected repeatedly in all 19 samples (core peptidome), peptide_mapper_script: the Perl script “peptide_mapper_script.txt” for mapping the peptides to their precursor proteins to extract different cleavage site features, peptide_mapper_script_manual.pdf: The Procedure for Peptide Mapping.

## Abbreviations

UPS	(Ubiquitin-Proteasome System)
PSM	(Peptide Spectrum Match)
FDR	(False Discovery Rate)
CTSD	(Cathepsin D)
MMP-2	(Matrix metalloproteinase-2)
