# Supplementary material for: Investigating and Annotating the Human Peptidome Profile from Urine under Normal Physiological Conditions
Source: Proteomes. 2024 Jun 25;12(3):18. doi: 10.3390/proteomes12030018 (PMC11270373; doi:10.3390/proteomes12030018)
Supplement: Supplementary file 1 [file proteomes-12-00018-s001.zip › proteomes-2985633-supplementary-7/peptide_mapper_script_manual.pdf]

The Procedure for Peptide Mapping

1. Preparation of Input Data

Prior to processing, ensure that the necessary input files are available. These include the following:

prtDB.fasta: This file comprises the protein database in FASTA format, containing the sequences to be mapped against the identified peptides.

peptide_list.txt: This file contains a list of identified peptide sequences derived from experimental data.

Both of these files are essential for successful peptide mapping and must be present in the directory where the peptide mapping script will be executed.

2. Installation of Perl

Before executing the peptide mapping script, ensure that Perl is installed on your system. Perl is required to run the script effectively. You can download and install Perl from the official Perl website (https://www.perl.org/) if it is not already installed on your system.

3. Execution of Peptide Mapping Script

Follow the steps below to execute the peptide mapping script:

1. Create Directory:

- Begin by creating a new directory to contain the downloaded peptide mapping script. Download “peptide_mapper_script.txt” and change the file extension to “peptide_mapper_script.pl".

- Ensure this directory also contains the input data files mentioned earlier.

2. Change Directory (cd):

- Open the command prompt.

- Navigate to the directory where the `peptide_mapper_script.pl` script and input files are located using the `cd` command followed by the path to the directory.

- Press Enter to confirm the directory change.

3. Start Processing:

- Execute the peptide mapping process by initiating the `peptide_mapper_script.pl` script.

- Enter the command to run the script in the command prompt, and then press Enter to start the processing.

Please note that the processing time may vary depending on the length of the peptide list being analyzed. For longer peptide lists, the processing duration may be considerable. It is recommended to exercise patience during this phase of the analysis.
